# Two-year follow-up of an open-label multicenter study of polyacrylamide hydrogel (Bulkamid®) for female stress and stress-predominant mixed incontinence

**DOI:** 10.1007/s00192-012-1761-8

**Published:** 2012-04-25

**Authors:** Philip Toozs-Hobson, Waleed Al-Singary, Michelle Fynes, Gunilla Tegerstedt, Gunnar Lose

**Affiliations:** 1 Department of Urogynaecology, Birmingham Women’s Hospital, Methchley Lane, Edgbaston, Birmingham B15 2TG UK; 2 Worthing Hospital, Department of Urology, Lyndhurst Road, Worthing, West Sussex BN11 2DH UK; 3 Department of Gynaecology, St George’s Healthcare NHS Trust, Blackshaw Road, Tooting, London, SW17 0QT UK; 4 Department of Obstetrics & Gynaecology, Karolinska University Hospital Huddinge, Hälsovägen, Flemmingsberg, 14186 Stockholm, Sweden; 5 Department of Obstetrics and Gynaecology, Herlev County Hospital, University of Copenhagen, Herlev Ringvej 75, 2730 Herlev, Denmark

**Keywords:** Bulking, Mixed incontinence, Stress, Polyacrylamide hydrogel, Bulkamid

## Abstract

**Introduction and hypothesis:**

Polyacrylamide hydrogel (PAHG, Bulkamid®) is a promising urethral bulking agent. This article presents the 2-year follow-up results of a multicenter study of PAHG injections for treating stress and stress-predominant mixed urinary incontinence.

**Methods:**

Submucosal injection of PAHG was performed in 135 women with urinary incontinence, with subjective and objective assessment of the efficacy and safety 24 months postinjection.

**Results:**

At 24 months, the subjective responder rate was 64 % (a statistically non-significant reduction from 67 % at 12 months). The decreased number of incontinence episodes and urine leakage were maintained compared with the result from the 12-month evaluations, as were objective result rates and quality of life data. No safety issues occurred.

**Conclusions:**

PAHG is an effective and safe treatment option for women with stress-predominant mixed urinary incontinence, with maintained medium-term responder rates.

## Introduction

Bulking agents were first described for treating stress urinary incontinence (SUI) more than 100 years ago [1] as a minimally invasive method to treat urinary incontinence (UI). Since that time, many bulking agents of different makes and tissue interactions have been used to treat UI. However, in the intervening period, many substances have been tried and abandoned due to concerns with both efficacy [2] and safety [3–9]. The mechanism of action of urethral bulking is not yet fully understood. One hypothesis is that intraurethral bulking produces coaptation of the urethral edges during the storage phase of the micturition cycle and particularly during periods of increased abdominal pressure [2]. Studies describing increased abdominal pressure transmission in the first quarter of the urethra [10] and increased abdominal leak-point pressure as a result of successful urethral injection outcomes [11] would support this hypothesis. Injectable agents therefore were thought to restore continence by increasing urethral resistance only at rest and allowing the urethra to funnel and open during micturition [12]. This theory was challenged by the publication of an alternative explanation of the mechanism of action [11, 13] whereby it was suggested that if positioned appropriately, the bulking agent could create a zone of increased contractility of the rhabdosphincter by creating an increased stretch of the muscle fibers. This theory proposed a possible mechanism whereby increase in the active component of the urethral pressure profile after bulking was explained. These two theories are not, of course, mutually exclusive, and there may be a combined mechanism.

The first description of the use of polyacrylamide hydrogel (PAHG) for urethral bulking was in 2006 [14]. Bulkamid® is a polymer gel consisting of 2.5 % cross-linked polyacrylamide and 97.5 % water for injection. It is atoxic [15, 16] and resistant to degradation [17, 18]. PAHG has been used in aesthetic plastic and reconstructive surgery in Europe for the past 7 years, and long-term as well as experimental studies show that the gel is gradually integrated through a fine network of vessel-bearing connective tissue, with no capsular fibrosis or calcification. Tissue integration starts immediately after implantation and is completed approximated 12-months post-injection depending on the bulk size [17–20]. The 12-month results of this study were previously presented, and they confirmed a 67 % subjective response rate, with a reduction in both incontinence episodes and 24-h pad weights [21]. The aim of the paper presented here was to assess the effectiveness and safety 2 years post PAHG injection in women with SUI or mixed UI (MUI) as a secondary endpoint to the initial presentation of the 1-year data.

## Materials and methods

The study was an open, noncomparative, multicenter, multinational study involving ten centers from five countries: two in Denmark, two in Sweden, one in Finland, four in the UK, and one in Germany. The materials and methods for this study were previously described in detail [21].

### Patient characteristics

Women aged ≥18 years with symptomatic SUI or MUI were eligible to participate. Inclusion criteria were duration of symptoms (≥12 months) and incontinence episode frequency (≥1 per 24 h). Additional requirements were a maximum flow rate of ≥15 ml/s, a postvoid residual urine volume (PVR) of ≤100 ml, a bladder capacity of ≥300 ml, and normal diuresis (<40 ml/kg per 24 h). Exclusion criteria were pelvic organ prolapse (POP) ≥stage 2; an acute urinary tract infection (UTI); allergic reaction to local anesthesia and antibiotics used for prophylaxis; previous surgery for incontinence, including bulking; ongoing medication for incontinence (except anticholinergics at a constant dose for >4 weeks); treatment with systemic corticosteroids; active autoimmune or connective tissue diseases; or pregnancy.

All women had a screening visit involving full medical history, physical and pelvic examination including genital prolapse (POP quantification) score, uroflowmetry, PVR measurement, and urine dipstick test (if positive with culture and sensitivity). The screening visit was followed by a baseline visit for collecting data from the 24-h pad-weighting test and 3-day micturition diary, including bladder capacity, number of micturitions, number of SUI and urge incontinence (UUI) episodes, and estimated renal output. Participants were also asked to complete the International Consultation on Incontinence Questionnaire (ICIQ) [21, 22] and a patient quality of life (QoL) and visual analogue scale (VAS) score [23]. Treatment with PAHG was performed at the baseline visit or within 3 days and no later than 8 weeks from the screening visit.

### Treatment and follow-up

PAHG injection was performed under local anesthesia (10 ml, 5 % lidocaine injected into the urethral wall). PAHG was injected under urethroscopic control transurethrally into the submucosa (three deposits of 0.2–0.8 ml each, 0.5–1 cm distal to the bladder neck) using a 23-gauge × 120-mm needle with 1-cm markings to ensure correct depth of injection placement. After satisfactory urethral occlusion, the bladder was emptied via the endoscope. During injection, the women received a single dose of prophylactic antibiotic treatment dependent on local protocol. Women were discharged after successful voiding (PVR <100 ml, assessed by bladder scanning). They were re-evaluated, as previously described [21], at 1 and 6 months, with 12-month follow-up as the primary endpoint. This paper presents the secondary endpoint of the extended 24-month follow-up. The 24-month visit efficacy assessment included patients’ subjective perception (cured, improved, not changed, or worsened) [24]. Objective assessment included 3-day micturition diary assessment for the number of incontinence episodes per 24 h and 24-h pad-weighting testing. Urine dipstick and PVR measurement were performed during the visit. QoL assessment was made by repeating the ICIQ and VAS score on QoL. Subjective responders were defined as women considering themselves as cured or improved. Women not responding to the initial injection were offered a second treatment between 6–8 weeks after the first injection. Adverse events (AE) were classified as serious or nonserious and judged to be either related (ADE) or unrelated (AE) to the study treatment.

### Data analyses and statistics

All analyses were carried out in identical fashion to those reported in the previous publication [21] and are therefore only described briefly. All analysis was based on the intention-to-treat (ITT) analysis set, which was defined as patients who received at least one treatment. ITT data were interpreted as described by Hollis and Campbell [25] and in keeping with other similar studies on bulking agents [26]. The sample size of 100 was estimated assuming a 50 % success rate, with a maximum of uncertainty of 10 %. Assuming a dropout rate of 20 %, a target of 125 participants was required. The primary end point for this calculation was the 12-month data previously reported [21]. This data set represents the secondary endpoint of 24 months. Detailed descriptions of the analysis on ICIQ scores, urine leakage (pad test), number of incontinence episodes, and VAS scores measuring QoL have previously been published [21].

### Additional analyses

Additional analyses were performed to evaluate the effect of other variables in the treatment outcome. Responder rate was assessed on data sets stratified by type of incontinence (stress vs mixed), number of treatments (one vs two), and number of procedures performed per center [<15 injections (low volume) vs ≥15 (high volume)]. As supportive analysis of the primary analysis, the logistic regression model was expanded by incorporating the covariates age and body mass index (BMI) as continuous variables in separate analyses.

### Ethical assurances

The study was performed in accordance with the International Conference on Harmonization Good Clinical Practice Guidelines; in accordance with the Declaration of Helsinki II, 1964, as amended in Scotland, October 2000; the Council Directive 93/42/EEC concerning medical devices; and the International Organization for Standardization/Draft International Standard (ISO/DIS) 14155-1 + 2:2003 Clinical investigation of medical devices for human subjects. The clinical investigation plan was submitted to all local ethics committees. Written consent was obtained from all patients after written and verbal information about the study, procedures, potential risk or inconveniences, and expected benefits.

## Results

One hundred thirty-five women (67 with SUI and 68 with MUI) were recruited and treated with PAHG. Patients’ demographic and incontinence-related characteristics were previously described [21]. Eighty-eight patients (65 %) had one treatment and 47 (35 %) received a second on request. The mean of total injected volume was 1.53 ml [standard deviation (SD) 0.48 ml], and the median volume per deposit was 0.5 ml. The median time spent on the injection procedure was 7 (range 2–20) min. Eighty-six women (64 % of the original cohort) were available for the 24-month follow-up. Of the withdrawals, one patient had an AE (aggravated UI), three withdrew consent, 30 reported lack of effect (eight went on to have further surgery, all being retropubic tapes), four previously successful patients were not included in the analysis because they withdrew >6 months before the 24-month assessment, and one patient was included as a responder who missed the 12-month analysis but attended the 2-year follow-up. One hundred and thirty-five patients were initially included in the intention to treat analysis. Following the rules described for last observation carried forward (LOCF) and imputation results for subjective outcome are presented for 124 patients at 12 months and 116 patients at 24 months. Table 1 shows the subjective success rates as both per protocol and intention to treat. On the intention to treat analysis at 24 months, the success rate was maintained at 64 % (which was a nonstatistically significant reduction from 67 % at 12 months). Twenty (17 %) were cured and 54 (47 %) improved. Of the responding patients at 12 months, 94 % (116/124) had sustained success rates at the 24-month follow-up.

**Table 1:** Subjective success rates at 1, 6, 12, and 24 months after treatment

	1 month	6 months	12 months	24 months
ITT (patients)	135	129	124	116
Responders %	87 %	71 %	67 %	64 %
CI	81–92 %	62–78 %	58–72 %	55–72 %

*ITT* intention to treat,* CI* confidence interval

**Fig. 1:**
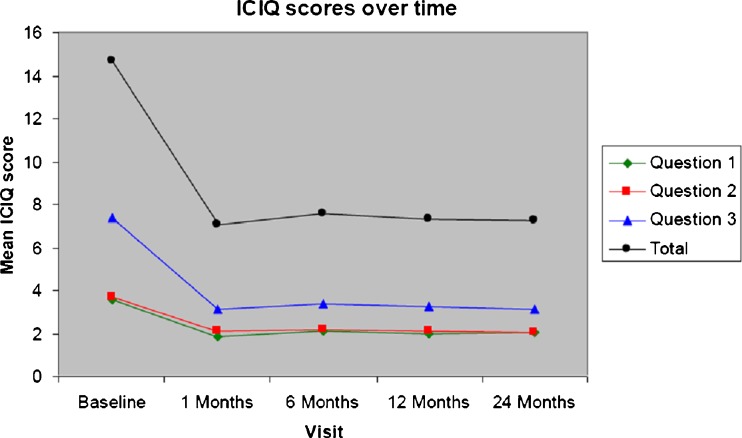
International Consultation on Incontinence Questionnaire (ICIQ) scores at 1, 6, 12, and 24 months [intention to treat (ITT) analysis]. Question 1: How often do you leak urine? (range 0–5). Question 2: How much urine do you usually leak? (range 0–6). Question 3: How much does leaking urine interfere with your everyday life? (range 0–10)

**Fig. 2:**
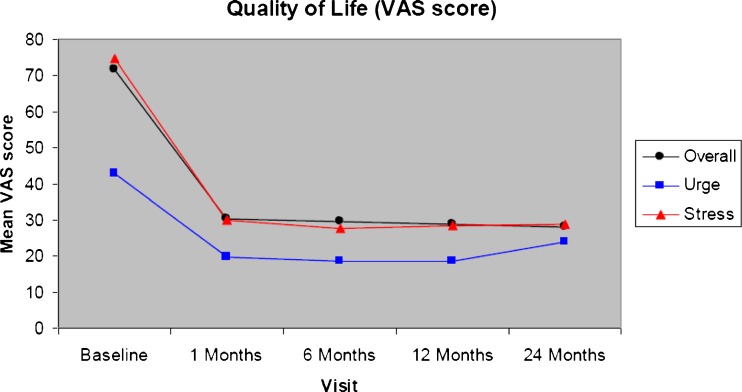
Patient quality of life visual analog scale (VAS) scores over timer in the intention to treat (ITT) analysis set

The slight deterioration seen with the subjective outcome is not recorded in either the ICIQ (Fig. 1) or VAS (Fig. 2) scores, where the improvement measured 12 months after treatment is maintained. Similarly, in the objective outcomes, both incontinence episode frequency and pad-weight test results retained the initial improvement. Results show a significant decrease in the number of incontinence episodes (*p* < 0.0001, Table 2) as well as a significant reduction in leakage from baseline to 24 months (*p* < 0.0001, Table 3). Consistent with the 12-month results, there was a nonstatistical trend toward a slightly better outcome for patients with “pure” SUI rather than MUI (69 % vs 58 %).

**Table 2:** Urinary leakage (pad test) over time

	Baseline	1 month	6 months	12 months	24 months
Mean leakage (g)					
Mean (SD)	54.3 (78.1)	20.8 (49.2)	14.6 (23.3)	10.9 (19.0)	13.2 (28.1)
Median	28.5	4.0	5.3	3.8	3.0

Leakage is the 24-h leakage measured as mean over 2 days

*SD* standard deviation

**Table 3:** Summary of daily incontinence episodes over time

	Baseline	1 month	6 months	12 months	24 months
Mean (SD)	3.68 (2.5)	1.78 (2.7)	1.81 (2.6)	1.55 (2.7)	1.53 (2.8)
Median	3.00	0.67	0.67	0.67	0.50

Daily number of incontinence episodes is the mean over 3 days registered in the patient’s diary

*SD* standard deviation

In terms of response to repeat treatment, the estimated responder rate was 69 % in patients receiving only one treatment and 53 % in patients receiving two treatments. Compared with the responder rates at 12 months follow-up (one treatment 72 %; two treatments 57 %), the differences were borderline statistically significant (*p* = 0.081)

### Safety evaluation

At the 24-month follow-up, 16 new nonserious AEs and four new serious adverse reactions were reported, none of which was thought to be related to the treatment. No woman had signs of impaired bladder emptying based on postvoid residuals at 24 months.

## Discussion

The evidence of medium/long-term durability and safety of different bulking agents is limited. We report the first systematic 2-year follow-up of PAHG (Bulkamid®) injection for SUI and MUI. The response rate of 67 % after 12 months (cured or improved) was sustained, which was supported by objective findings of 24-h pad-weighting test, number of incontinence episodes, and QoL data. Furthermore, no safety issues occurred between 1 and 2 years’ follow-up. The findings are in good agreement with a study by Ghoniem et al. that showed 88 % durability success and few complications after 2 years [27]. In contrast, two studies on transurethral injection of hyaluronic acid/dextranomer (NASHA/Dx gel) showed poor durability of effect and a high incidence of complications [28, 29].

PAHG and Macroplastique® (silicone-based) are nonresorbable and nondegradable bulking agents. After injection with these gels, the foreign-body reaction is mild (PAHG) to modest (Silicone). Silicone, however, is hydrophobic and has a tendency to migrate from the implantation site via circulating phagocytic cells [30, 31] in contrast to PAHG, which is hydrophilic. Moreover, PAHG will interact with the surrounding tissue as a consequence of the high water content and structure and integrate into the surrounding tissue through a fine network of vessel-bearing connective tissue, thus being firmly anchored into the surrounding tissue. Analysis of cost effectiveness suggests that the decline in the use of bulking agents may be premature and that there may be a valid economic argument to consider their use [32]. Advantages of bulking agents are ease of application, which can be achieved in the office setting, and a reduced side effect/complication risk for patients, where this is the more pressing part of the risk–benefit ratio.

This study, as with many others, has continued to supply evidence demonstrating that PAHG in pure SUI tend to achieve better outcomes than those for MUI [14, 33]. However, as with other surgeries, this treatment method may lead to improvement in QoL as a result of decreased symptoms, rather than cure, and as such may have a valuable therapeutic role within stress-predominant MUI. In our previous paper [21], the potential mechanisms of action were discussed. No attempt at site assessment or implant action was made during this study, and so no conclusion can be made as to the mode of action. It would seem logical, given the length of the urethra, that both mechanisms may be involved, and further assessment of placement site and success may be of value.

Safety concerns have been raised regarding specific bulking agents because of migration, hypersensitivity, urethral erosion, pseudocysts/abscess, granuloma formation, and obstruction [3–9, 28, 29]. All AEs recorded after PAHG injection were generic and not related to the material. No treatment-related AEs were reported by the investigators during follow-up from 12 to 24 months.

Indications for using bulking agents to treat UI remain a contentious issue. In one publication, the cure rate of a repeat midurethral sling was significantly lower and the incidence of de novo urgency and UUI was significantly higher in repeat procedures [34]. Urethral bulking may therefore be an alternative to repeat midurethral sling procedures.

## Conclusions

This report presents the largest series in a powered study demonstrating medium-term longevity of PAHG treatment of UI, with no trend toward a decrease in efficacy at 2 years. The reason for this is almost certainly related to the properties of the polyacrylamide implant, in which the bulking substance is made up predominantly of water held within the polyacrylamide gel matrix. The substance is unlikely to migrate, and indeed, there are no reported cases of this in any of the applications in medicine in over 20 years of use. Secondly, there are no reported AEs, such as granuloma or abscess formation, suggesting that the substance remains inert, does not metabolize, and as such maintains shape and size. We therefore conclude that these properties are central to the results achieved in this series 2 years posttreatment. These results confirm PAHG to be an easy and safe bulking agent for treating uncomplicated SUI or MUI, with favorable durability 2 years after injection.
